# Trehalose, an mTOR Independent Autophagy Inducer, Alleviates Human Podocyte Injury after Puromycin Aminonucleoside Treatment

**DOI:** 10.1371/journal.pone.0113520

**Published:** 2014-11-20

**Authors:** Yu-Lin Kang, Moin Ahson Saleem, Kwok Wah Chan, Benjamin Yat-Ming Yung, Helen Ka-Wai Law

**Affiliations:** 1 Department of Health Technology and Informatics, Faculty of Health and Social Sciences, The Hong Kong Polytechnic University, Hunghom, Hong Kong, China; 2 Department of Nephrology and Rheumatology, Shanghai Children's Hospital, Shanghai Jiao Tong University, Shanghai, China; 3 Academic Renal Unit, University of Bristol, Southmead Hospital, Bristol, United Kingdom; 4 Department of Pathology, Li Ka Shing Faculty of Medicine, The University of Hong Kong, Queen Mary Hospital, Pokfulam, Hong Kong, China; UCL Institute of Child Health, United Kingdom

## Abstract

Glomerular diseases are commonly characterized by podocyte injury including apoptosis, actin cytoskeleton rearrangement and detachment. However, the strategies for preventing podocyte damage remain insufficient. Recently autophagy has been regarded as a vital cytoprotective mechanism for keeping podocyte homeostasis. Thus, it is reasonable to utilize this mechanism to attenuate podocyte injury. Trehalose, a natural disaccharide, is an mTOR independent autophagy inducer. It is unclear whether trehalose alleviates podocyte injury. Therefore, we investigated the efficacy of trehalose in puromycin aminonucleoside (PAN)-treated podocytes which mimic cell damage in minimal change nephrotic syndrome *in vitro*. Human conditional immortalized podocytes were treated with trehalose with or without PAN. Autophagy was investigated by immunofluorescence staining for LC3 puncta and Western blotting for LC3, Atg5, p-AMPK, p-mTOR and its substrates. Podocyte apoptosis and necrosis were evaluated by flow cytometry and by measuring lactate dehydrogenase activity respectively. We also performed migration assay to examine podocyte recovery. It was shown that trehalose induced podocyte autophagy in an mTOR independent manner and without reactive oxygen species involvement. Podocyte apoptosis significantly decreased after trehalose treatment, while the inhibition of trehalose-induced autophagy abolished its protective effect. Additionally, the disrupted actin cytoskeleton of podocytes was partially reversed by trehalose, accompanying with less lamellipodias and diminished motility. These results suggested that trehalose induced autophagy in human podocytes and showed cytoprotective effects in PAN-treated podocytes.

## Introduction

Glomerular diseases are characterized by the disrupted renal filtration barrier which consists of endothelial cells, basement membrane and epithelial cells (also called podocytes). Podocytes have been regarded as the key target of harmful stimuli in renal diseases [1]. It is evident that podocyte effacement is commonly found in glomerular diseases such as minimal change nephrotic syndrome (MCNS) and focal segmental glomerulosclerosis (FSGS) [2]. In addition, the gene mutation of podocyte cytoskeleton proteins is associated with congenital nephrotic syndrome [3]–[5]. Thus, the ideal therapeutic strategies of glomerular diseases are aiming to ameliorate podocyte injury including apoptosis and actin cytoskeleton rearrangement.

Autophagy has emerged as a potential approach recently. It is a highly conserved catabolic mechanism of which the unwanted organelles and misfolded proteins are delivered to lysosome for degradation [6]. The final metabolic products can be recycled as nutrient for keeping cell homeostasis. Podocytes are postmitotic and long-lived cells. Therefore, strong self-protective mechanisms are required for counteracting different detrimental challenges. As expected, the higher basal autophagy has been demonstrated in podocytes than other renal cells [7], [8]. Autophagy deficient mice present with accumulated dysfunction proteins, endoplasmic reticulum stress and proteinuria. Moreover, they are more susceptible to drug-induced models of glomerular diseases [8]. Our previous study also showed that inhibition of autophagy increases apoptosis and actin cytoskeleton depolymerisation in puromycin aminonucleoside (PAN)-treated human podocytes [9]. Additionally, it was reported that autophagy may become as a new therapeutic approach for diabetic nephropathy [10]. Collectively, it is suggested that autophagy may be harnessed for the treatment of glomerular diseases, as it may attenuate podocyte injury by eliminating harmful stimuli [6], [11].

Autophagy is precisely regulated by a network of proteins. For instance, mTOR which is a highly conserved serine/threonine kinase negatively regulates autophagy induction [12]. Rapamycin is the representative agent for inducing autophagy via inhibition of mTOR activity. It was demonstrated that autophagy induced by rapamycin alleviates podocyte injury *in vitro* [13]. However, proteinuria was observed in patients with organ transplantation after rapamycin treatment [14], and the underlying mechanism remains unclear. Even though the beneficial effects of autophagy in renal diseases are not fully realized, it is essential to find alternative safe autophagy inducers for the treatment of renal diseases.

Trehalose is a natural disaccharide found in organisms from bacteria to plants, including yeast, fungi, and invertebrates [15], [16]. It induces autophagy in an mTOR-independent manner and shows protective effects in various cells against harmful stimuli such as heat, dehydration, cold, desiccation, and oxidation [17]. The protective effect of trehalose has been demonstrated in neurodegenerative diseases, such as Alzheimer's disease, frontotemporal dementia, progressive supranuclear palsy and corticobasal degeneration [17], [18]. It remains unclear whether trehalose induces autophagy and shows cytoprotective effects in podocytes.

In the present study, we are the first to show that trehalose induced autophagy in an mTOR independent manner in human podocytes. Moreover, podocyte apoptosis and actin cytoskeleton depolymerisation can be alleviated by trehalose. Therefore, this study confirmed that autophagy can be utilized for protecting podocytes and trehalose may be a potential candidate for the treatment of glomerular diseases.

## Methods And Materials

### Cell culture

Conditionally immortalized human podocytes AB8/13 (University of Bristol, UK) were cultured in RPMI 1640+10% fetal bovine serum (Life Technologies, New York, USA) [19]. The human podocytes proliferated at non-permissive temperature (33°C) with the addition of Insulin-Transferrin-Selenium (Life Technologies). Cells were harvested after growing to 50–60% confluency and passaged to permissive temperature (37°C) for full differentiation in 10–14 days. Fully differentiated human podocytes were used in the following experiments. Agents for podocyte treatment included trehalose (10–100 mM, optimum at 50 mM), PAN (30 µg/ml), chloroquine (CQ, 25 µm) and wortmannin (WT, 0.2 µm) (Sigma-Aldrich, Missouri, USA). The dose of both autophagy inhibitors has been optimized in dosage titration experiments (data not shown).

### Western blotting

Total proteins in podocytes were extracted by RIPA buffer containing phosphatase inhibitors (Calbiochem, Merck Millipore, USA) and protein inhibitors (Roche Applied Science, Mannheim, Germany). Bradford assay (Bio-Rad Laboratories, Munich, Germany) was used to assess protein concentration. Equal amounts of proteins were denatured after heating at 95°C for 5 min and separated by 4–12% SDS-PAGE. PVDF membrane (Bio-Rad) after transferring was incubated with primary antibody and secondary antibody. Eventually the results were visualised using chemiluminescence substrate (Perkin Elmer, Massachusetts, USA). The ChemiDoc MP system (Bio-Rad) was used to capture images. The expression of proteins was analysed with the Image J software. Rabbit anti-human LC3, Atg5, p-mTOR, T-mTOR, p-p70S6K, p70S6K, p-4E-BP1, 4E-BP1, p-AMPK, AMPK, p62, GAPDH and goat anti-rabbit IgG-HRP linked antibody (Cell Signaling Technology, Massachusetts, USA) have been used with a 1∶1000 dilution.

### Immunofluorescence analysis

Fixed podocytes (4% paraformaldehyde, 15 min) were blocked with 2% BSA for 30 min. After washing in PBS three times, podocytes were incubated with anti-LC3 antibody (4°C, 12 h). Alexa Fluor 488 goat anti-rabbit IgG antibody (Life Technologies) was added (room temperature, 2 h) and washed. ProLong Gold Antifade Reagent with DAPI (Life Technologies) was used to stain nuclei. Cells were visualised by confocal microscopy (EZ-C1, Nikon Instrument, Japan). Furthermore, Alexa Fluor 594 Phalloidin (Life Technologies) was used for Filamentous actin (F-actin) staining. The percentage of podocytes showing accumulation of LC3 puncta (that is with at least five puncta per podocyte) were counted and podocyte with disrupted cytoskeleton was quantified as the previous study described [9], [20]. The lamellipodias per cell were calculated following the method described before [21]. At least 100 cells were scored in each of the six independent experiments.

### Reactive oxygen species (ROS) measurement

Podocytes were cultured in RPMI 1640 free of phenol red (Life Technologies) in black 96-well plates (Perkin Elmer). Differentiated podocytes were incubated with 10 µM CM-H_2_DCFDA (Life Technologies) or vehicle alone (20 min), followed with trehalose treatment (50 mM). After recovering for 0.5 hour (37°C), immunofluorescence was recorded using the VICTOR 3 plate reader (Perkin Elmer). The fluorescence intensity was calculated and analysed as described previously [22].

### Necrosis measurement

Equal number of podocytes was seeded in 6-well plates. Podocyte necrosis was evaluated by measuring lactate dehydrogenase (LDH) activity in culture medium. The CytoTox 96 Non-Radioactive Cytotoxicity Assay (Promega, Wisconsin, USA) was used for assessing LDH activity. The manufacture's protocol was strictly followed [23].

### Migration assay

Fully differentiated podocytes in 6-well plates were scratched by two strokes with a sterile 0.4 mm 200 µl Gilson style extension length tip. Podocytes were treated by PAN and/or trehalose for 12 h. The number of podocytes migrating into the gap was counted as described before [23].

### Apoptosis measurement

Podocytes were harvested after treatment and the cell density was adjusted to 1×10^6^/ml. (1) YO-PRO-1/PI assay (Life Technologies). 1 ml of podocytes suspension was incubated with 1 µl YO-PRO-1 and 1 µl PI solution for 30 min on ice. Apoptotic cells were measured by flow cytometer (FC500, Beckman Coulter, California, USA) and expressed as a percentage of total cells. (2) Anti-active caspase-3 assay. PE-conjugated active caspase-3 antibody (BD Pharmingen, California, USA) was used to stain podocytes which were pre-incubated with Cytofix/Cytoperm solution. Cells were resuspended in BD Perm/Wash buffer and the percentage of active caspase-3 positive podocytes was analysed by flow cytometry. Examples of plots and analysis are shown in our previous study [9].

### Statistical Analysis

Data were expressed as mean ± SEM. T-test was used for two group's comparison. Multiple groups were analysed by Kruskal-Wallis test with post hoc procedures using the Prism 5.0 Software (GraphPad, San Diego, CA, USA). A *p* value <0.05 was considered to be statistically significant. n denotes the number of independent experiments performed.

## Results

### Trehalose induced autophagy in human podocytes

**Figure 1:**
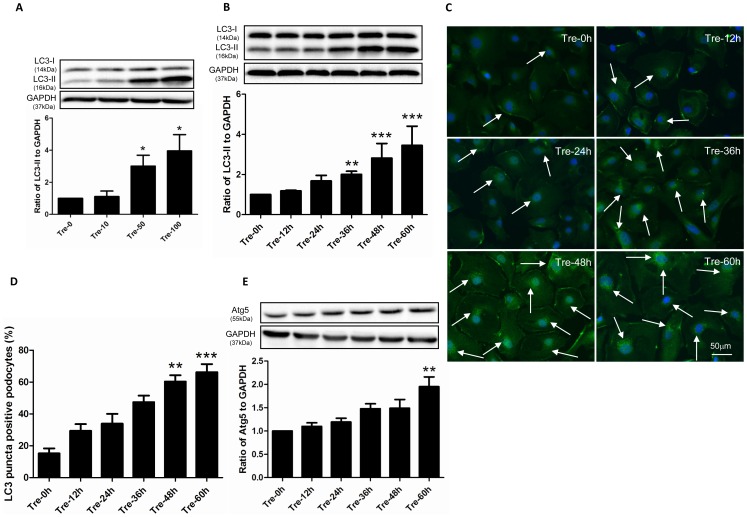
Trehalose induced autophagy in human podocytes. (**A**) The expression of LC3-II increased in a dosage dependent manner. Conditionally immortalized human podocytes were treated with 0, 10, 50 and 100 mM of trehalose (Tre) for 48 h. LC3-II was measured by Western blotting. The data (means ± SEM) was expressed as the relative changes compared with Tre-0 mM group. Representative immunoblot images were shown along with the statistical results. *p<0.05 versus Tre-0 mM, n = 5. (**B**) LC3-II increased in a time dependent manner. Podocytes were treated with 50 mM Trehalose for 0, 12, 24, 36, 48 and 60 h. **p<0.01, ***p<0.001 versus Tre-0 h, n = 7. (**C–D**) LC3-II puncta increased after trehalose treatment. LC3 immunostaining in podocytes was performed at 0, 12, 24, 36, 48 and 60 h after trehalose treatment (50 mM). Significant increased green bright puncta (indicated by white arrows) can be observed in cytoplasm after 48 h-trehalose treatment. The representative LC3 immunostaining images were shown along with statistical results from 6 independent experiments. **p<0.01, ***p<0.001 versus Tre-0 h. Podocyte nuclei were stained with DAPI (blue). (**E**) The expression of Atg5 was up-regulated in trehalose-treated podocytes (50 mM). Podocytes were treated with 50 mM Trehalose for 0, 12, 24, 36, 48 and 60 h. The expression of Atg5 significantly increased at the time point of 60 h. **p<0.01 versus Tre-0 h, n = 5.

To test whether trehalose induces autophagy in human podocytes, we investigated the expression of LC3 and Atg5 in treated cells. Briefly, LC3 consists of LC3-I and LC3-II, LC3-I converts into LC3-II which adheres to the membrane of phagophore for the formation of autophagosome. LC3-II presents with bright puncta in immunofluorescence staining [24]. Atg5 is essential for the elongation of autophagosome [25]. As shown in Figures 1A–1B, the expression of LC3-II increased in a dosage and time dependent manner, the immunofluorescence staining results were consistent with these findings. The LC3-II puncta positive podocytes significantly increased in the cytoplasm after 36 h-trehalose treatment (Figures 1C–1D). In addition, the significant increased expression of Atg5 was found at the time point of 60 h (Figure 1E).

### Trehalose induced podocyte autophagy in an mTOR independent manner

**Figure 2:**
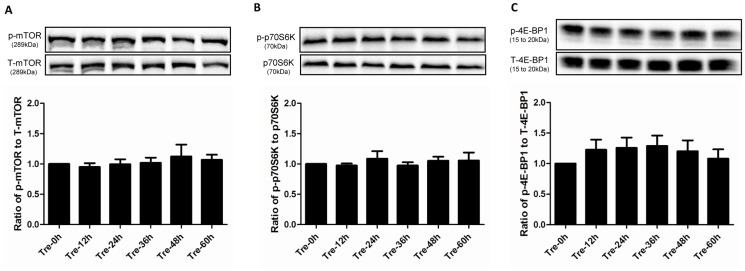
Trehalose induced podocyte autophagy in an mTOR independent manner. mTOR activity was not altered in trehalose-treated podocytes. Podocytes were treated with trehalose (50 mM) for 0, 12, 24, 36, 48 and 60 h. The phosphorylation level of p-mTOR (**A**) and its substrates p-p70S6K (**B**) and p-4E-BP1 (**C**) were measured by Western blotting, n = 5, 5 and 6 respectively. However, no significant changes were observed. The representative immunoblot was shown along with the statistical results.

mTOR negatively regulates autophagy induction, whereas trehalose triggers autophagic flux in other cell types such as neuron without altering the phosphorylation level of phospho-mTOR (p-mTOR) [17]. It remains unknown whether the same pathway occurs in trehalose-treated podocytes. We measured the phosphorylation level of p-mTOR and its substrates p-p70S6K, p-4E-BP1. As shown in Figures 2A–2C, these three markers did not changed significantly over the 60 h treatment.

### Trehalose-induced autophagy was independent of ROS

**Figure 3:**
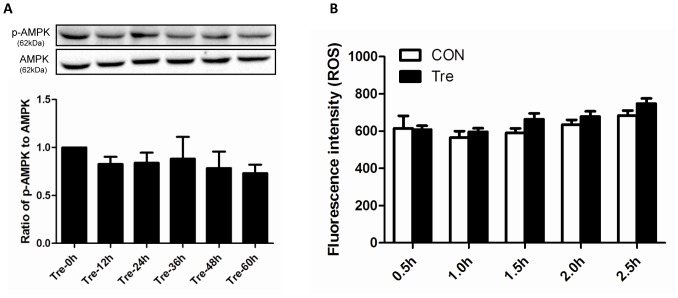
Trehalose-induced podocyte autophagy was independent of ROS. Trehalose-induced autophagy was not associated with energy restriction and ROS. (**A**) Trehalose-treated podocytes (50 mM) were harvested for p-AMPK measurement at the time points of 0, 12, 24, 36, 48 and 60 h. The phosphorylation level of p-AMPK did not change significantly. n = 6. (**B**) ROS level was recorded every half an hour after trehalose treatment (50 mM), the data representing immunofluorescence intensity within 2.5 h were shown (n = 6). No significant changes were noted.

Starvation is the most common stimulus for inducing autophagy. To verify whether trehalose-induced autophagy attributes to calorie limitation, we examined the phosphorylation level of p-AMPK which is an energy sensor in mammalian cells. When the ratio of AMP to ATP decreases, p-AMPK will increase responsively and subsequently lead to autophagy induction by inhibiting the activity of mTOR [26]. As shown in Figure 3A, no significant changes existed in phosphorylation level of p-AMPK. The underlying mechanism of starvation-induced autophagy is the generation of ROS which is essential for the initiation of autophagic flux [27]. Therefore, to confirm whether ROS is involved in the trehalose-induced autophagy, we monitored the ROS production. Consistently, ROS did not change significantly when compared with control group (Figure 3B).

### Trehalose decreased PAN-induced apoptosis in human podocytes via the induction of autophagy

**Figure 4:**
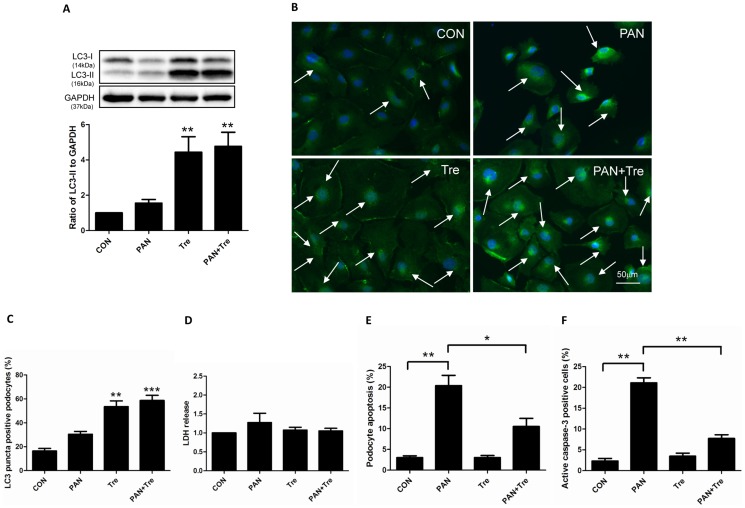
Trehalose decreased PAN-induced apoptosis in human podocytes. Podocyte were treated with PAN (30 µg/ml) or/and Trehalose (50 mM) for 48 h. (**A**) Trehalose induced autophagy in PAN-treated human podocytes. The expression of LC3-II slightly increased after 48 h PAN treatment, while it dramatically up-regulated in Tre and PAN+ Tre groups. Representative immunoblot images were shown along with the statistical results. **p<0.01 versus CON, n = 6. (**B–C**) The findings of (A) were confirmed by LC3 immunostaining. Obvious elevated LC3-II bright green puncta (indicated by white arrows) were visualized in trehalose-treated groups (Tre and PAN+ Tre groups), the representative images and statistical results were shown. Nuclei were stained in blue. **p<0.01, ***p<0.001 versus CON, n = 6. (**D**) No significant changes were observed in podocyte necrosis. LDH in culture medium of 4 groups was measured, n = 4. (**E**) Elevated apoptosis in PAN-treated podocytes was decreased by trehalose. Apoptosis was measured by flow cytometry with YO-PRO-1/PI assay. Podocyte apoptosis was induced by PAN and decreased significantly by trehalose. *p<0.05, **p<0.01 versus CON, n = 8. (**F**) The findings of (E) were confirmed by the data of active caspase-3 measurement. The active caspase-3 positive podocytes were measured by flow cytometry. The changes pattern was similar to podocyte apoptosis measured by YO-PRO-1/PI assay. **p<0.01 versus CON, n = 7.

Interplay exists between autophagy and apoptosis. It has been reported that autophagy suppresses cell apoptosis by inhibiting apoptotic proteins [28]. Since trehalose can induce autophagy in podocytes without increasing ROS, we hypothesized that trehalose decreases podocyte apoptosis. PAN was used to induce podocyte apoptosis *in vitro*. We found that trehalose up-regulated the expression of LC3-II in PAN-treated human podocytes (Figure 4A). The data of LC3 immunostaining confirmed this finding as LC3-II bright puncta were obviously presented in trehalose alone and PAN+ Tre groups (Figures 4B–4C). To confirm the protective effects of trehalose, LDH was measured to evaluate podocyte necrosis. As shown in Figure 4D, the changes in LDH level was negligible. However, PAN-induced apoptosis was significantly down-regulated after trehalose treatment, accompanying with the decrease in active caspase-3 positive cells (Figures 4E–4F).

**Figure 5:**
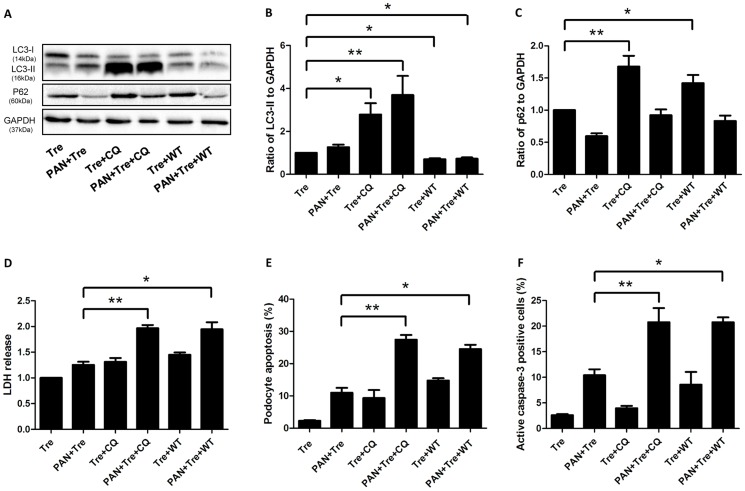
Inhibition of trehalose-induced autophagy abolished its cytoprotective effects in preventing podocyte apoptosis. CQ (25 µM) or WT (0.2 µM) was used to inhibit podocyte autophagy which was induced by trehalose (50 mM) or trehalose (50 mM) + PAN (30 µg/ml) for 48 h. (**A–C**) CQ and WT inhibited trehalose-induced autophagy. The expression of LC3-II drastically increased in Tre+CQ and PAN+ Tre+CQ groups, while it decreased significantly in Tre+WT and PAN+ Tre+WT groups. p62 slightly decreased in PAN+ Tre group, whereas it significantly increased in Tre+CQ and Tre+WT groups. The immunoblot images were shown along with statistical data. *p<0.05, **p<0.01 versus Tre group, n = 7. (**D**) Necrosis increased after the inhibition of trehalose-induced autophagy. The LDH in culture medium was measured. *p<0.05, **p<0.01 versus PAN+Tre group, n = 6. (**E**) Podocyte apoptosis increased after the inhibition of trehalose-induced autophagy. The percentage of apoptotic podocytes was much higher in PAN+Tre+CQ and PAN+Tre+WT groups than the PAN+ Tre group. *p<0.05, **p<0.01 versus PAN+ Tre group, n = 7. (**F**) The percentage of active caspase-3 positive podocytes increased after inhibition of trehalose-induced autophagy. The changes in the percentage of active caspase-3 positive podocytes were similar to the data in (E). *p<0.05, **p<0.01 versus PAN+ Tre group, n = 8.

To verify whether the cytoprotective effects of trehalose are attributed to autophagy induction, chloroquine (CQ) and wortmannin (WT) were used to inhibit autophagy. CQ blocks the fusion between autophagosome and lysosome, resulting in an accumulation of LC3-II. WT, a potent and specific phosphatidylinositol 3-kinase (PI3-K) inhibitor, inhibits autophagy at the initial stage with the decreased LC3-II [29]. As shown in Figures 5A-5B, CQ increased the expression of LC3-II in trehalose-treated groups. Meanwhile, WT showed the opposite pattern. In order to confirm the inhibitory effect of CQ and WT, we also measured the expression of p62 (also named SQSTM1) which oligomerizes polyubiquitinated proteins and binds to LC3 on the autophagosome membrane [30]. Eventually, these labelled damaged organelles or unfolded proteins will be degraded in the autophagy pathway. Thus, the inhibition of autophagy should result in p62 accumulation. As shown in Figure 5C, the expression of p62 was up-regulated when trehalose-induced autophagy was inhibited by CQ or WT. This pattern of expression was also observed when CQ or WT were added to the PAN+ Tre group (PAN+ Tre+CQ and PAN+ Tre+ WT group respectively). LDH increased significantly when trehalose-induced autophagy was being inhibited (Figure 5D). Correspondingly, podocyte apoptosis increased after CQ or WT treatment as well as the percentage of active caspase-3 positive podocytes (Figures 5E–F).

### Trehalose alleviated PAN-induced actin cytoskeleton injury in human podocytes

**Figure 6:**
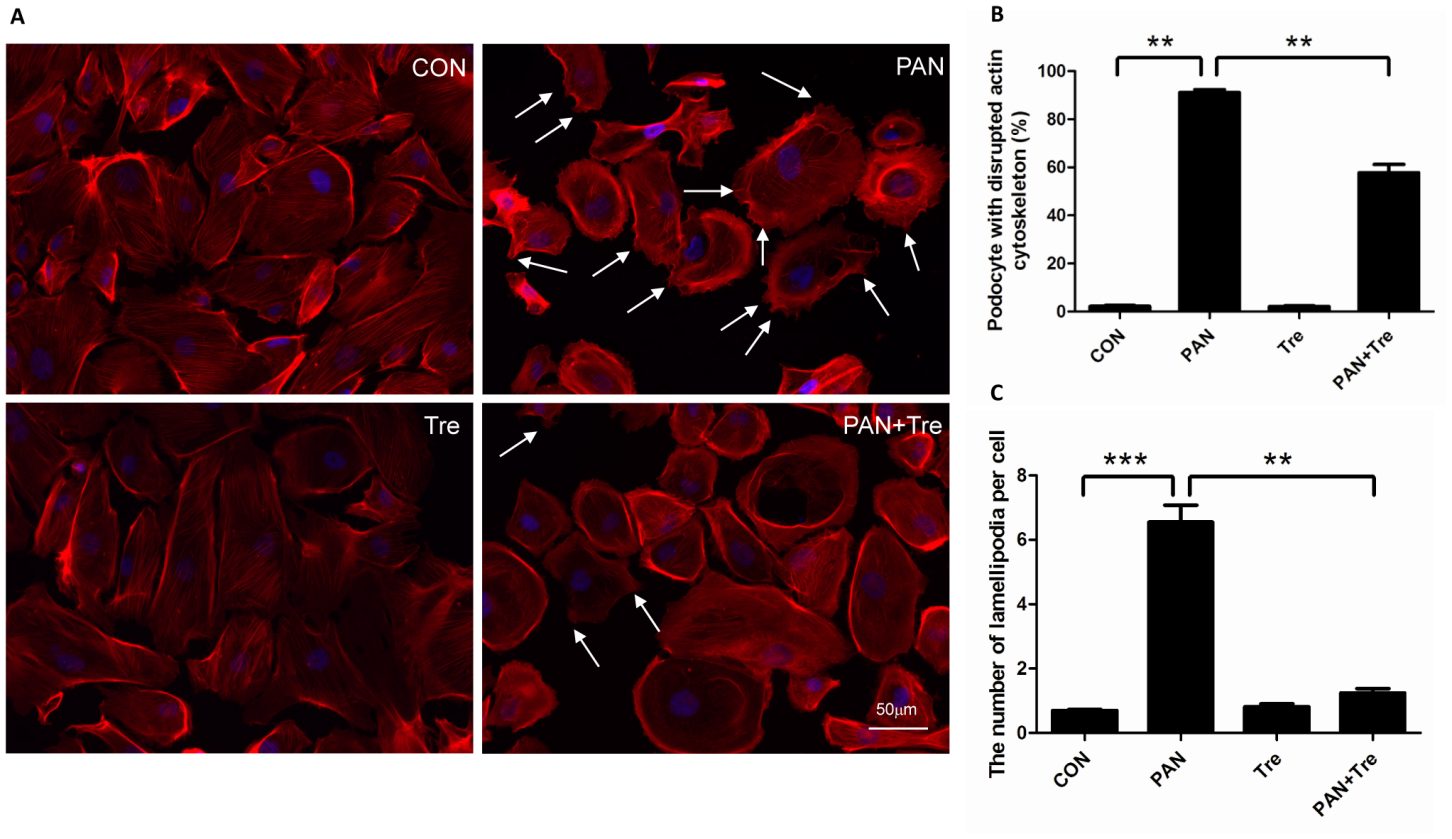
Trehalose alleviated PAN-induced actin cytoskeleton injury in human podocytes. Podocyte were treated with PAN (30 µg/ml) or/and Trehalose (50 mM) for 48 h. (**A**) F-actin was stained for evaluating the integrity of actin cytoskeleton. PAN induced actin cytoskeleton depolymerisation which was characterized by irregular distribution of F-actin. Meanwhile, plenty of lamellipodias were formed (indicated by white arrows). The actin cytoskeleton damages were partially reversed by trehalose. The representative F-actin staining images were shown. (**B–C**) PAN-induced actin cytoskeleton damage in podocytes was alleviated by trehalose. The number of PAN-treated podocytes with disrupted actin cytoskeleton was down-regulated by trehalose, accompanying with decreased lamellipodias. **p<0.01, ***p<0.001 versus CON, n = 6.

The actin cytoskeleton is the major mechanical support for maintaining the integrity and contractility of podocytes [1]. PAN-treated podocytes are characterized by the disrupted actin cytoskeleton and generation of lamellipodia (Figure 6A). After trehalose treatment, the percentage of podocytes with disrupted actin cytoskeleton decreased as well as the number of lamellipodias (Figure 6B–6C).

### Trehalose diminished cell motility in PAN-treated human podocytes

**Figure 7:**
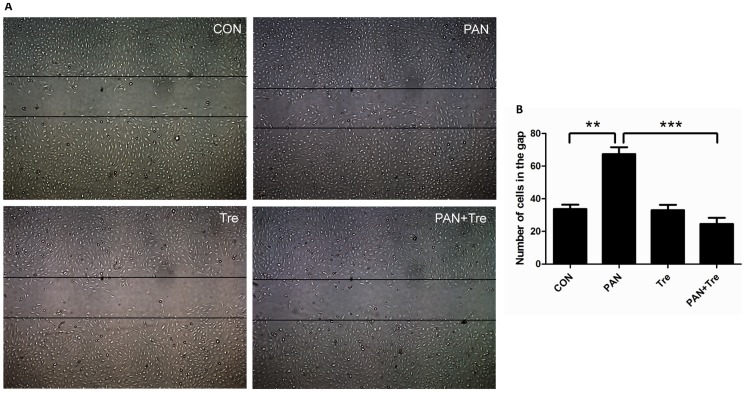
Trehalose diminished cell motility in PAN-treated human podocytes. Podocyte were treated with PAN (30 µg/ml) or/and Trehalose (50 mM) for 12 h. (**A**) PAN-increased podocyte motility was suppressed by trehalose. The representative images were taken under inverted microscope (50×). (**B**) The number of cells migrating into the gap was calculated for evaluating podocyte motility. The migrated podocytes after PAN treatment increased significantly, whereas trehalose suppressed this enhanced motility. **p<0.01, ***p<0.001 versus CON, n = 6.

The limited physiological motility is essential for keeping podocytes in normal function. Either enhanced or low level motility of podocytes is detrimental and considered to be the cause of proteinuria [1]. As shown in Figures 7A–7B, PAN-treated podocytes migrated faster than control, whereas the enhanced motility was decreased to the normal level after trehalose treatment.

## Discussion

Glomerular capillary is encircled by foot processes which withstand high filtration pressure. Podocyte damage is regarded as the determining factor of glomerular diseases [31]. Thus, alleviating podocyte injury becomes the therapeutic aim. High basal level of autophagy has been demonstrated in podocytes *in vitro* and *in vivo*, suggesting that autophagy plays a vital role in promoting podocyte survival [7], [8]. Theoretically, autophagy induction may ameliorate podocyte damage and autophagy inducers may be developed as new therapeutic agents for renal diseases. Here, we showed that PAN-induced podocyte injury was alleviated by trehalose.

Trehalose is regarded as the autophagy inducer in neurological studies. Moreover, the safety of trehalose is well recognized [32]. Therefore, we investigated whether trehalose provided benefits to the damaged podocytes. Firstly, we revealed that trehalose induced autophagy in human podocytes. The expression of LC3-II and Atg5 in podocytes increased after trehalose treatment in a dosage and time dependent manner. The results of LC3 immunostaining also demonstrated that elevated LC3-II puncta were formed in cytoplasm. Hence, trehalose is an autophagy inducer in human podocytes and may be a potential agent for the treatment of glomerular diseases.

mTOR is one of the most important cellular negative regulators of autophagy [33]. Our data showed that trehalose did not significantly alter the phosphorylation level of p-mTOR and its substrates including p-p70S6K and p-4E-BP1. Therefore, in accordance with the results of trehalose studies in other disciplines such as neurology [34], our data elucidated that trehalose induced autophagy in an mTOR independent manner in human podocytes. To date, several mTOR independent pathways have been discovered. For instance, lithium, carbamazepine or valproic acid induce autophagy by decreasing inositol via the inositol signalling pathway [35]. L-type Ca^2+^ channel antagonists such as verapamil, loperamide, amiodarone, nimodipine and nitrendipine enhance autophagy by activating the Ca^2+^/Calpain pathway [36]. In addition, the cAMP/Epac/Ins (1, 4, 5) P_3_ pathway was also reported [37]. However, it is still unknown which pathway is involved in trehalose-induced autophagy. Thus, more studies are needed to explore the underlying mechanisms.

ROS has been regarded as a common signalling molecular for autophagy induction. For instance, starvation induces autophagy via the production of ROS which specially regulates the activity of Atg4 [27]. However, trehalose did not promote the generation of ROS, suggesting no induction of cell stress in podocytes. Consistently, it has been reported that ROS does not change after trehalose treatment in HeLa cells [38]. In addition, our study showed that the phosphorylation level of p-AMPK did not change significantly, and no sign of the activation of AMPK was detected. Therefore, our results suggested that trehalose induced autophagy without changing the energy status and it may have fewer side effects than other autophagy inducers.

In the present study, we used PAN-treated podocytes to test the efficacy of trehalose, as this model is characterized by the increased apoptosis and actin cytoskeleton rearrangement mimicking podocyte damage in glomerular diseases [9]. We found that PAN slightly increased the expression of LC3-II at 48 h, suggesting autophagy was also induced under cell stress, but it was kept at a low level at this time point. Additionally, trehalose can enhance autophagy in PAN-treated podocytes. As a result, PAN-induced podocyte apoptosis was decreased by trehalose. This finding was also confirmed by the percentage of active caspase-3 positive podocytes. Furthermore, two autophagy inhibitors (CQ and WT) based on different principles were used for verifying the role of trehalose-induced autophagy. The expression pattern of LC3-II and p62 revealed that trehalose-induced autophagy was blocked, but it may be puzzling that p62 was not elevated in PAN+Tre+CQ and PAN+Tre+WT groups. The possible reason is that p62 was partially degraded in advance by PAN which also induced a low level of autophagy in podocytes at 48 h. Consequently, necrosis increased and the decreased apoptosis by trehalose rebounded after autophagy inhibition. Taken together, it suggested that trehalose decreased podocyte apoptosis via autophagy induction. In future, more studies may be carried out to explore how trehalose-induced autophagy decreases podocyte apoptosis. As it has been reported that autophagy can decrease apoptosis by inhibiting the production of active caspase 8 and tBid [39]. Moreover, autophagy eliminated dysfunctional mitochondria to decrease the generation of cytochrome C which activates apoptosis [40].

Actin cytoskeleton depolymerisation was another important feature of glomerular diseases [41]. We showed that trehalose partially reversed actin cytoskeleton reorganization and suppressed the formation of lamellipodia which was associated with cell motility. It is well known that podocytes are motile cells, but the motility is kept at an optimal level. The enhanced motility is detrimental to podocytes and is responsible for podocyte effacement *in vivo* [1]. The data of migration assay demonstrated that PAN accelerated podocyte migration, whereas trehalose decreased this enhanced motility to the normal level. Taken together, it is suggested that trehalose also protected podocytes by maintaining the stability and normal motility of actin cytoskeleton. However, it is difficult to verify whether the cytoprotective effect in podocyte actin cytoskeleton is attribute to trehalose-induced autophagy, because autophagy inhibitors (CQ and WT) disrupted actin cytoskeleton to similar extend as PAN alone at 48 h (data not shown). Additionally, it has been reported that autophagy inhibition (Atg7 knockout) lead to actin rearrangement in mouse embryonic fibroblast cells [42]. Thus, our data showed that trehalose can protect actin cytoskeleton in podocytes, but the underlying mechanisms need to be investigated in the future.

In conclusion, trehalose induced autophagy and alleviated podocyte injury including apoptosis and actin cytoskeleton depolymerisation. Notably, trehalose may be safer than other autophagy inducers, not only because it is a natural disaccharide widespread throughout the biological world, but also it induced autophagy independent of mTOR and ROS in human podocytes. As mTOR is involved in diverse cell functions such as protein synthesis, ribosome biogenesis and cell cycle, trehalose theoretically produces fewer side effects. On the contrary, rapamycin is a strong autophagy inducer due to the inhibition of mTOR, and it may partially explain why rapamycin achieves remission in podocyte injury *in vitro* but it leads to proteinuria in patients with renal transplantation [33]. Moreover, trehalose activated autophagic flux without causing cell stress in human podocytes. Therefore, we proposed that autophagy induction is a novel strategy for the treatment of glomerular diseases and trehalose is a good candidate for inducing autophagy in podocytes. In future studies, the efficacy of trehalose could be tested in PAN nephrosis rat and adriamycin nephropathy model. Hopefully, trehalose can be applied to patients with MCNS and FSGS for alleviating podocyte injury. However, autophagy is a double edged sword. Over-activation of autophagy or prolonged autophagy induction may lead to cell death. In future, the questions of how to minimise this risk and how to precisely regulate autophagy induction need to be addressed for autophagic therapy.
